# Monitoring-Based Rewards Enhance Both Learning Performance and Metacognitive Monitoring Accuracy

**DOI:** 10.3390/bs15030307

**Published:** 2025-03-05

**Authors:** Shaohang Liu, Christopher Kent, Josie Briscoe

**Affiliations:** 1 Institute of Developmental Psychology, Faculty of Psychology, Beijing Normal University, Beijing 100875, China; 2 Beijing Key Laboratory of Applied Experimental Psychology, National Demonstration Center for Experimental Psychology Education, Beijing Normal University, Beijing 100875, China; 3 School of Psychological Science, University of Bristol, Bristol BS8 1TU, UK; c.kent@bristol.ac.uk (C.K.); j.briscoe@bristol.ac.uk (J.B.)

**Keywords:** reward-based learning, metacognitive monitoring, performance-related anxiety, intrinsic motivation

## Abstract

Utilization of monetary rewards in educational settings remains contentious due to its potential adverse effects such as performance-related anxiety, metacognitive inaccuracy, and diminished intrinsic motivation. The current study developed a novel reward-based learning paradigm wherein rewards are granted based on monitoring accuracy rather than learning performance. Specifically, learners receive rewards for items that they predict they will remember and subsequently successfully remember them during the final test. Two experiments were conducted to assess the efficacy of this paradigm: Experiment 1 focused on learning Chinese medicine images, while Experiment 2 examined the transfer of math knowledge in classroom settings. The results indicated that rewarding the alignment between performance and metacognitive accuracy improved learning performance compared to both a baseline group and a group receiving performance-based rewards. Furthermore, this paradigm effectively mitigated performance-related anxiety and preserved intrinsic motivation. Overall, our findings highlight the critical role of reward-based learning design and emphasize the importance of addressing metacognitive accuracy alongside performance in educational practice.

## 1. Introduction

It is well documented that monetary incentives enhance task performance (e.g., Musslick et al., 2015; Umemoto & Holroyd, 2015). In educational contexts, providing students and children with monetary reward is popular to enhance their learning. However, the mechanisms underlying reward-based learning are complex. Monetary rewards can induce some positive effects, such as increasing attention and enhancing dopamine release in the hippocampus (Deci et al., 2001; Montague et al., 1996; Murayama et al., 2010), as well as some negative effects, including reducing intrinsic motivation (Deci et al., 1999), and provoking performance-related anxiety (Benware & Deci, 1984; Vansteenkiste et al., 2004; Lepper et al., 2005). Typically, the use of monetary rewards in real classroom settings is discouraged due to their potential negative effects. However, it remains an intriguing question whether a paradigm for providing rewards could be designed to avoid these adverse outcomes. In this study, we proposed a novel approach to reward-based learning: incentivizing metacognitive monitoring accuracy rather than learning performance.

## 2. Incentive Rewards and Learning Performance

Although motivation is considered a core factor influencing learning and memory (Deci et al., 1991; Dweck, 1986), the role of incentive rewards in learning and memory remains mixed. Providing performance-based rewards can have a direct positive effect by activating the mesolimbic reward system, which promotes release of dopamine in the hippocampus and facilitates memory consolidation (Knowlton & Castel, 2022). However, many studies have demonstrated various negative effects of monetary rewards on learning and memory. For instance, performance-based rewards can provoke performance-related anxiety and pressure, impairing learning outcomes (e.g., Ellis & Ashbrook, 1989). When students are motivated to perform optimally, their cognitive processes can be disrupted by irrelevant thoughts, such as worrying about poor performance or doubting their abilities, which hinders deep processing of study materials (Baumeister, 1984; DeCaro et al., 2011). Additionally, studies have shown that when learning behaviors are driven by extrinsic rewards, the level of perceived anxiety triggered by performance-based rewards may lead to a more superficial learning style characterized by shallow levels of elaboration and less effortful encoding (e.g., Benware & Deci, 1984; Simons et al., 2004; Vansteenkiste et al., 2004; Lepper et al., 2005).

Aside from this, extrinsic rewards can undermine students’ intrinsic motivation for learning (Deci et al., 1999). This undermining effect is evidenced by decreased satisfaction and poor learning performance when a non-reward learning activity follows a reward-based one. Additionally, studies have shown that people’s motivation for choosing challenging tasks to perform may be offset by the risk of losing out potential extrinsic rewards. Murayama and Kuhbandner (2011) even found that the beneficial effect of incentive rewards only worked for the content that participants were not interested in, but disappeared for the content that they were interested in.

The mixed effects of performance-based rewards create a dilemma. On one hand, providing monetary rewards appears to be an easy-to-implement and time-efficient technique to enhance learning performance. On the other hand, monetary rewards might produce a set of negative influences. Given these considerations, an important question arises: Is there a design of reward-based learning that can enhance learning performance and also avoid negative effects of performance-based rewards?

## 3. Non-Performance-Based Rewards

A primary concern with using incentive rewards in educational settings is their typical dependence on students’ learning or academic performance. To address this concern, decoupling rewards from performance may offer noticeable benefits (Fröber & Dreisbach, 2016, 2020). For example, Westbrook et al. (2013) reported a paradigm that measures participant’s subjective cost of cognitive effort, where participants chose between low-effort tasks for smaller rewards and high-effort tasks for larger rewards. The additional compensation required to equalize these choices quantified the subjective cost of cognitive effort. The study demonstrates that cognitive effort discounting correlates with task load, performance, and individual traits like the need for cognition. For example, older adults perceived cognitive tasks as more effortful and required significantly higher compensation than younger adults, even when performance was controlled. This suggests that providing greater rewards for increased mental effort may counteract the decline in motivation (Kool et al., 2013; Shenhav et al., 2017).

Recently, Lin et al. (2024) introduced an innovative reward-based paradigm targeting to enhance individuals’ willingness to choose challenging tasks to perform by providing them rewards based on the level of difficulty of the tasks they choose to performance, rather than based on their task performance. In this study, participants in a performance group received rewards according to their task performance, whereas those in an effort group received rewards solely based on the level of difficulty of the tasks they chose to perform. After the intervention phase, both groups were invited to choose a new task among a set of tasks with different levels of difficulty to perform. The results showed that effort-based rewards increased participants’ willingness to choose challenging tasks to perform, and this enhancement effect even successfully transferred to subsequent tasks in which the rewards were completely removed. Interestingly, despite that the effort group was selected to perform more difficult tasks than the performance group, overall task performance did not differ between these two groups, suggesting that non-performance-based rewards (e.g., effort-based rewards) could effectively redefine the role of incentives in enhancing task performance.

In the current study, we developed a novel reward paradigm that shifts the focus from rewarding learning performance to rewarding metacognitive monitoring accuracy. Specifically, learners are required to make a judgment of learning (JOL, i.e., estimating the likelihood of remembering a study item in a later test) after studying each item. Rewards are structured such that learners receive the highest reward for successfully remembering the items they predict they will remember, a reduced reward for successfully remembering the items they predict they will forget, and a modest reward for accurately predicting the items that are forgotten. No reward is given for the items that they predict they will remember but actually forget. It is worth noting that, in this reward paradigm, learners still receive higher rewards for items they successfully remember than for the items they forget. This design is implemented to prevent the risk that some learners may choose to not study any items and predict they will forget all items in order to obtain maximum rewards (see below for detailed discussion).

## 4. Rationale of Monitoring-Based Rewards

We assume that monitoring-based rewards should offer noticeable benefits and address some drawbacks of performance-based rewards in three keyways.

First, individuals can benefit from making JOLs and rewarding JOL accuracy. On the one hand, previous studies showed that making JOLs can enhance learning performance, a phenomenon known as the *JOL reactivity effect* (Janes et al., 2018; Li et al., 2022, 2024; Zhao et al., 2022). Although the reactivity effect of JOL has only been found for the related word pairs, studies have noted the beneficial mechanisms of making JOLs in improving learning. For example, the *enhanced-engagement theory* suggests that the requirement of making JOLs for each item can sustain learners’ attention and improve overall learning performance by preventing attentional waning (Tauber & Witherby, 2019; Tekin & Roediger, 2020; Zhao et al., 2022). The *cue-strengthening theory* posits that learners need to search for “diagnostic” cues to guide JOL formation, and the cue-searching process in turn induces deeper level-of-processing of the study items and therefore enhances learning performance (Soderstrom et al., 2015). On the other hand, monitoring-based rewards can improve learning performance by enhancing metacognitive monitoring accuracy. Studies have shown that students with higher metacognitive monitoring accuracy tend to achieve better learning performance (Metcalfe & Finn, 2008; Van Loon & Oeri, 2023), especially during self-regulated learning. This correlation is likely because students with higher metacognitive monitoring accuracy can more appropriately allocate their cognitive resources (Bjork et al., 2013), engage in deeper processing of the learning material (Toppino & Cohen, 2010), and adaptively adjust their learning strategies (Bjork et al., 2013).

Second, monitoring-based rewards can mitigate some negative effects (e.g., performance-related anxiety) associated with performance-based rewards. It has been well-shown that performance-related anxiety can disrupt cognitive processing and impede elaborative processing (Baumeister, 1984; DeCaro et al., 2011). This anxiety can also negatively affect metacognitive monitoring (Janes et al., 2018; Halamish & Undorf, 2022; Rivers et al., 2021), and the learner may fail to effectively allocate their study resources, such as study time. We assume the monitoring-based reward can reduce the anxiety caused by performance-based reward. On the one hand, by decoupling rewards from performance, monitoring-based rewards should be able to reduce performance-related anxiety by eliminating fear of poor performance, as such rewards are not contingent upon successful retrieval (Ashcraft & Krause, 2007). Furthermore, monitoring-based rewards are expected to enhance students’ sense of control, as they receive greater rewards for items they believe they have mastered, thereby alleviating performance anxiety (Bandura & Cervone, 1983; Lachman & Weaver, 1998). Additionally, studies have shown that focusing more on intrinsic improvement (i.e., what knowledge is acquired) can reduce the anxiety caused by peer pressure (DeCaro et al., 2011). Making Judgments of Learning (JOLs) encourages students to reflect on the knowledge they have learned, rather than focusing on external factors such as “how much money is lost”.

Third, different from performance-related rewards, monitoring-based rewards should not undermine students’ intrinsic learning motivation. According to the *cognitive evaluation theory* (CET), extrinsic rewards can diminish intrinsic motivation by undermining feelings of self-control and competence (Lepper, 1973). This theory argues that rewards for engaging in interesting activities may lead individuals to attribute their behaviors to external rewards rather than their intrinsic interests. However, by aligning rewards with metacognitive monitoring accuracy rather than test performance, students are encouraged to reflect on their actual level of knowledge mastery rather than focusing solely on how well their final test performance will be. This approach may help maintain intrinsic motivation by emphasizing the value of accurate self-assessment over mere performance outcomes.

## 5. Overview of the Current Study

In the current study, we aimed to explore whether aligning rewards with metacognitive monitoring accuracy rather than test performance can enhance learning outcomes and meanwhile mitigate some drawbacks associated with performance-based rewards. To investigate this, we employed four groups of participants, inducing (a) a performance reward group, where participants’ final rewards were based on their test performance, (b) a monitoring reward group, where participants’ final rewards were based on their metacognitive monitoring accuracy, (c) a monitoring group, where participants made item-by-item JOLs without receiving any rewards related to monitoring accuracy, and (d) a baseline group, where participants did not make JOLs and did not receive any rewards related to monitoring accuracy and test performance. The performance reward group was not asked to make judgments of learning (JOLs) because our aim was to compare the monitor reward group with the most conventional method of providing rewards—where the amount of reward is determined solely by performance.

Based on the preceding discussions, we formulated three hypotheses: (a) learning performance should be higher in the monitoring reward group than in the other three group; (b) performance-related anxiety should be lower in the monitoring reward group than in the performance reward group; and (c) intrinsic motivation to learn new information should be higher in the monitoring reward group than in the performance reward group.

## 6. Experiment 1

Experiment 1 was conducted in a laboratory to test the three hypotheses discussed above.

### 6.1. Methods

#### 6.1.1. Participants

As no previous studies have ever explored related effects, we did not conduct power analysis based on previously reported effect size. A power analysis performed via G*Power (Faul et al., 2007) suggested a minimum sample size of 45 participants in each group to observe a moderate effect size (Cohen’s *f* = 0.25) at 0.80 power. Due to over-recruitment, 220 undergraduate students from a Chinese university were recruited as participants. Nine participants did not return to complete the final test and their data were excluded from analyses, leaving final data from 211 participants (*M*_age_ = 20.1, *SD* = 1.6; 100 female). There were 53 participants in the baseline group, 51 in the monitoring group, 54 in the performance reward group, and 53 in the monitoring reward group.

All participants signed an agreement to participate. They obtained monetary reward after completion of the experiment. Specifically, participants from the baseline and monitoring group received 40 RMB at the end of the second-day test. For the two reward groups, participants obtained 40 RMB for their participation and a bonus dependent on their test performance (performance reward group) or metacognitive monitoring accuracy (monitoring reward group). Note that we did not control the total amount of reward across the four experimental groups, as our aim was to investigate whether the provision of additional rewards can increase learning performance, and how this design of reward-based learning can be optimized (see Table 1 for descriptive statistics of reward amount of four experimental groups). The protocol was ethically approved by the Faculty of Psychology, Beijing Normal University.

#### 6.1.2. Material

The learning materials were 40 Chinese medicine images. Each image were 400 × 400 pixels with its name presented below. The materials have been made publicly available at OSF (https://osf.io/3czqr/?view_only=e7d6ea6f9e174b06b794d660e4b1814e, accessed on 16 May 2024). All stimuli were presented via Psychopy v2023.1.2 (Peirce, 2007).

#### 6.1.3. Procedure

The experiment consisted of two sessions: a study session and a test session. Participants were instructed that they would learn 40 Chinese medicine images and take a final test on the following day. In the performance reward group, participants were further informed that they would earn 2 RMB for each item successfully recalled in the final test. In the monitoring reward group, participants were asked to predict whether they would be able to remember the name of each Chinese medicine image after 24 h. They were told that they would earn: (a) 2 RMB for the items that they predicted they would remember and actually successfully remembered in the final test, (b) 0.5 RMB for the items that they predicted they would forget but actually remembered in the final test, (c) 1 RMB for the items that they predicted they would forget and actually forgot in the final test, and (d) 0 RMB for the items that they predicted they would remember but actually forgot in the final test (see Table 1 for average reward obtained for the performance reward group and monitoring reward group). Note that only when participants try their best can they obtain the most reward. The purpose of setting differential amount of reward for condition (a) and (c) is to avoid the possibility that participants just skip the item.

In the monitoring group, participants were only asked to make JOLs and did not receive any instructions about monetary rewards. The baseline group did not receive any instructions about monetary rewards and JOLs, and was solely asked to learn and memorize the image–name pairs.

During the study session, participants learned the image–name pairs one-by-one in a random order. Each pair was presented for 10 s with a 1-s interstimulus interval inserted between each pair of study items. The image was displayed at the center of the screen with its name shown beneath it. In the baseline and performance reward groups, participants viewed the image–name pair for 10 s without making JOLs. In the monitoring and monitoring reward groups, a JOL question appeared beneath the image–name pair asking, “*Will you be able to remember the name of this image after 24 h?*” Participants indicated their metacognitive judgments by clicking on either the “*WILL*” or “*WILL NOT*” button. If no response was made during 10 s, an exclamation mark reminder appeared during the interstimulus interval after the image trial.

After studying all image–name pairs, participants rated their anxiety level using a 9-point Likert scale (“*Please rate the anxiety level during learning the images. 1 = not anxious at all*; *9 = very anxious*”). Then participants were dismissed and invited to come back for the final test 24 h later. In the final test administered 24 h later, the 40 medicine images were presented one-by-one in a random order, and participants were asked to recall the name of each medicine image displayed. There was no time pressure and no feedback in the final test.

Following the final test, participants were invited to participate in an additional task involving learning another 40 Chinese medicine images using the same study-test procedure but without any rewards. Specifically, they were told that “*There will be another chance for you to learn additional 40 images with the same procedure, and you will not get any monetary rewards for this learning task. Please rate your willingness to participate in this task, and our experimenter would contact you if you are willing to participate*.” They were asked to rate their willingness on a 9-point Likert scale (*1 = not willing at all*; *9 = very willing*). This question was implemented to measure participants’ intrinsic motivation, and explore whether monitoring-based rewards can avoid reducing intrinsic motivation induced by performance-based rewards.

### 6.2. Results

**Figure 1:**
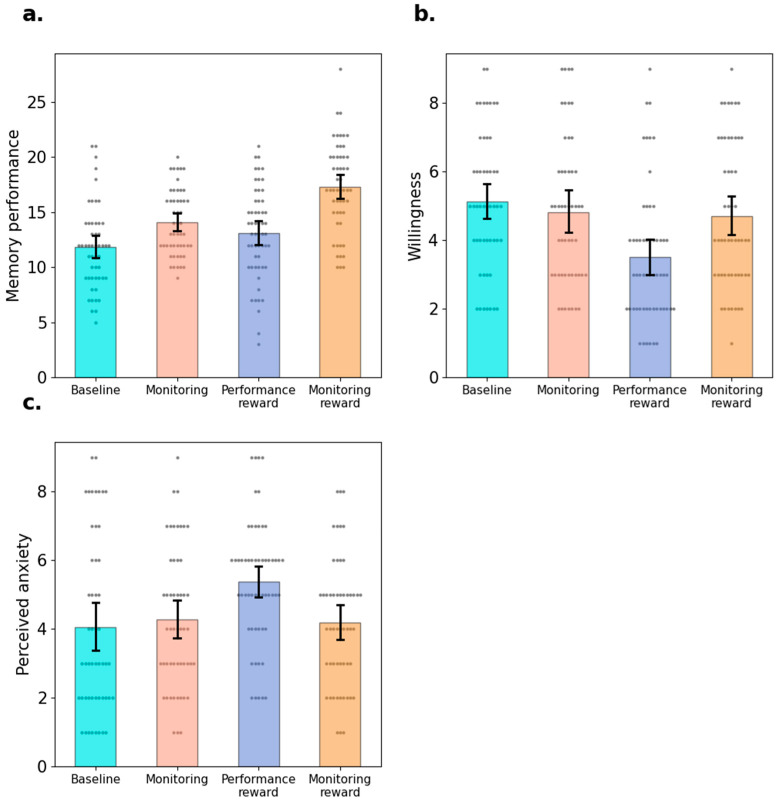
Results of Experiment 1. *Note.* Memory performance (Panel **a**), willingness to participate in the subsequent task without reward (Panel **b**) and test anxiety (Panel **c**) of four groups. Error bars indicate 95% confidence intervals and the grey dots represent the value of individual participants.

A one-way ANOVA was conducted to compare the memory performance of four experimental groups. Significant differences were detected: *F*(3,207) = 19.63, *p* < 0.001, $\eta_{p}^{2}$ = 0.22, *BF*_10_ > 1000 (see Figure 1a). Post hoc comparisons, using Bonferroni corrected *t*-tests, demonstrated that the monitoring reward group (*M* = 17.26, *SD* = 4.06) achieved best test performance, and test performance in this group was significantly better than that in the monitoring group (*M* = 14.04, *SD* = 3.00), difference = 3.23 [1.30, 5.15], *t* = 4.34, *p* < 0.001, *d* = 0.851, *BF*_10_ > 1000, higher than that in the performance reward group (*M* = 13.11, *SD* = 4.12), difference = 4.15 [2.21, 6.09], *t* = 5.42, *p* = 0.001, *d* = 1.092, *BF*_10_ > 1000, and higher than that in the baseline group (*M* = 11.84, *SD* = 3.83), difference = 5.42 [3.50, 7.35], *t* = 7.05, *p* < 0.001, *d* = 1.43, *BF*_10_ > 1000. Additionally, the monitoring group successfully recalled more items compared with the baseline group, difference = 2.20 [0.25, 4.14], *t* = 2.93, *p* = 0.02, *d* = 0.58, *BF*_10_ = 18.81. There was no detectable difference in final test performance between the baseline and performance reward groups, difference = 1.26 [−0.64, 3.16], *t* = 1.72, *p* = 0.52, *d* = 0.33, *BF*_10_ = 0.68, and no detectable difference between the performance reward and monitoring groups, difference = 0.93 [−0.97, 2.83], *t* = 1.27, *p* = 0.21, *d* = 0.25, *BF*_10_ = 0.45.

As shown in Figure 1b, the ANOVA shows that the four groups reported differential motivations to participate in the subsequent task in which rewards were no longer provided, *F*(3,207) = 6.57, *p* < 0.001, $\eta_{p}^{2}$ = 0.09, *BF*_10_ = 78.98. Bonferroni corrected *t*-tests showed that participants in the performance reward group (*M* = 3.52, *SD* = 1.99) reported lower motivation than the baseline group, difference = −1.64 [−2.67, −0.60], *t* = −4.10, *p* < 0.001, *d* = −0.79, *BF*_10_ = 418.84, lower than the monitoring group, difference = −1.32 [−2.36, −0.29], *t* = −3.31, *p* = 0.006, *d* = −0.64, *BF*_10_ = 25.34. and lower than the monitoring reward group difference = 1.20 [−2.22, −0.17], *t*= −3.03, *p* = 0.015, *d* = −0.58, *BF*_10_ = 12.06. However, there were no differences in motivation between the baseline and the monitoring group, difference = 0.31 [−0.75,1.37], *t* = 0.77, *p* = 0.89, *d* = 0.15, *BF*_10_ = 0.27, and between the monitoring group and monitoring reward group, difference = 0.13 [−0.92, 1.17], *t* = 0.31, *p* = 0.89, *d* = 0.06, *BF*_10_ = 0.22.

As is shown in Figure 1c, the ANOVA shows that the levels of anxiety experienced during the learning phase differed significantly among the four groups, *F*(3,207) = 4.69, *p* = 0.003, $\eta_{p}^{2}$ = 0.06, *BF*_10_ = 7.85. Specifically, Bonferroni corrected t-tests showed that the performance reward group (*M* = 5.38, *SD* = 1.82) reported higher levels of anxiety compared with the baseline group (*M* = 4.04, *SD* = 2.57), difference = 1.34 [0.29, 2.38], *t* = 3.30, *p* = 0.007, *d* = 0.64, *BF*_10_ = 14.48, higher than the Monitoring group (*M* = 4.28, *SD* = 2.05), difference = 1.10 [0.05, 2.15], *t* = 2.58, *p* = 0.04, *d* = 0.53, *BF*_10_ = 9.05, and higher than the monitoring reward group (*M* = 4.19, *SD* = 1.87), difference = 1.19 [0.15, 2.22], *t* = 2.82, *p* = 0.02, *d* = 0.57, *BF*_10_ = 27.29. However, no difference was found between the baseline and the monitoring groups, difference = 0.24 [−0.84,1.31], *t* = 0.57, *p* = 1.00, *d* = 0.11, *BF*_10_ = 0.24., and between the monitoring and monitoring reward groups, difference = 0.09 [−0.98, 1.15], *t* = 0.21, *p* = 1.00, *d* = 0.04, *BF*_10_ = 0.21.

Metacognitive monitoring accuracy for each participant in the monitoring and monitoring reward groups was measured as the Gamma (*G*) correlation between predicted performance and actual performance. Metacognitive monitoring accuracy in the monitoring reward group (*M* of *G* = 0.32, *SD* = 0.68) was significantly higher than that in monitoring group (*M* = 0.02, *SD* = 0.66), difference = 0.30 [0.04, 0.56], *t*(102) = 2.30, *p* = 0.02, *d* = 0.45, *BF*_10_ = 2.14, suggesting that monetary rewards successfully enhanced monitoring accuracy.

### 6.3. Discussion

Experiment 1 confirmed the effectiveness of monitoring-based rewards, demonstrating its superior benefits compared to other learning strategies or interventions, such as monitoring-only and traditional performance-based rewards. Additionally, monitoring-based rewards do not undermine intrinsic motivation and do not provoke performance-related anxiety as performance-related rewards do.

## 7. Experiment 2

Experiment 2 aimed to evaluate the effectiveness of monitoring-based rewards in real educational settings and assess its transferability to different learning tasks. To achieve this, Experiment 2 employed a different participant sample (high school students) and used a different type of learning materials (mathematical theorems).

### 7.1. Method

#### Participants

The sample size was not pre-determined. Instead, it was determined by the number of available classes and students in each class. In total, 246 grade 10 (high school) students from 4 classes in a high school were recruited initially, while 6 students did not attend the second-day task and hence were excluded, leaving final data from 240 students (*M*_age_ = 16.3, *SD* = 0.51; 129 female). Students from four classes were randomly assigned to four experimental groups. The size of each group was determined by the size of the class, so there were 60 students in each class and literally in each experimental group.

The academic performance of four classes (grades taken from students’ mid-term exam) were well matched. Likewise Experiment 1, all participants obtained 40 RMB for participation at the end of the experiment, and two reward groups obtained a bonus dependent on their test performance (performance reward group) or metacognitive accuracy (monitoring reward group).

### 7.2. Materials

The learning materials comprised 10 mathematical theorems, including concepts such as *the Cosine Theorem*, which the students had not previously encountered. These theorems were presented in a ten-page format, with each page containing one theorem along with an example of its application (see OSF https://osf.io/3czqr/?view_only=e7d6ea6f9e174b06b794d660e4b1814e for the materials, accessed on 16 May 2024).

### 7.3. Procedure

Experiment 2 was conducted in a classroom setting, following a similar study-test procedure as that in Experiment 1, but adapted for an educational environment. Participants were instructed that they would learn 10 mathematical theorems, each accompanied by an example, and they would take a final test the following day. In the final test, participants were required to apply the theorems to solve novel problems. As in Experiment 1, participants were informed about the reward structure specific to their experimental group (see Table 1 for average reward obtained for the performance reward group and monitoring reward group).

During the learning session, all students received learning materials with a blank cover. They began reading the materials once the “begin” instruction was given by the teacher. Unlike Experiment 1, where the study phase was experimenter-paced, the study phase in Experiment 2 was self-paced. That is, in Experiment 2, students were allowed to self-regulate their time across a total of 30 min for studying the whole set of materials. For participants in the monitoring and monitoring reward groups, they needed to answer “*Can you master this theorem in the test after 24 h?*” by ticking “*CAN*” or “*CAN’T*” on each page.

To maintain ecological validity, participants were not asked to report their anxiety levels after the learning session. On the second day, participants completed a final test consisting of ten questions, where they applied the learned theorems to solve novel problems. They were given 45 min to complete the test. After finishing the final test, participants were asked about their willingness to participate in a similar task, with the same learning procedure but without any monetary rewards. They rated their willingness using a 9-point Likert scale (1 = *not willing at all*; 9 = *very willing*).

## 8. Results

**Figure 2:**
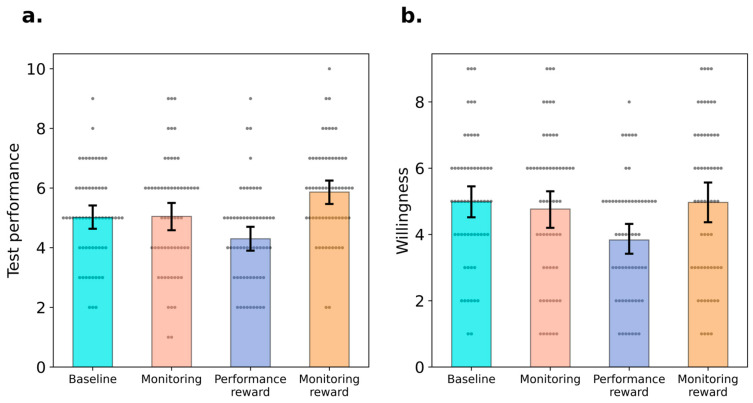
Results of Experiment 2. *Note.* Test performance (Panel **a**), willingness to participate in the subsequent task without reward (Panel **b**) of four groups. Error bars indicate 95% confidence interval and the grey dots represent the value of individual participants.

A one-way ANOVA was conducted to compare the test performance between four experimental groups. There was a significant difference across four groups (Figure 2a), *F*(3, 236) = 8.39, *p* < 0.001, $\eta_{p}^{2}$ = 0.10, *BF*_10_ > 1000. Specifically, Bonferroni corrected *t*-tests showed that the monitoring reward group (*M* = 5.87, *SD* = 1.58) achieved better performance compared with baseline group, difference = 0.85 [0.07, 1.64], *t* = 2.80, *p* = 0.03, *d* = 0.51, *BF*_10_ = 10.11, monitoring group, difference = 0.82 [0.03, 1.60], *t* = 2.70, *p* = 0.04, *d* = 0.49, *BF*_10_ = 3.54, and performance reward group (*M* = 4.30, *SD* = 1.60), difference = 1.57 [0.78, 2.35], *t* = 0.11, *p* < 0.001, *d* = 0.94, *BF*_10_ > 1000. However, no difference was found between monitoring group (*M* = 5.05, *SD* = 1.91) and baseline group (*M* = 5.02, *SD* = 1.53), difference = 0.03 [−0.75, 0.82], *t* = 0.11, *p* = 0.91, *d* = 0.02, *BF*_10_ = 0.20. Performance of the performance reward group was numerically worse than the baseline group, difference = −0.72 [−1.50, 0.07], *t* = −2.36, *p* = 0.11, *d* = −0.43, *BF*_10_ = 3.18, and the monitoring group, difference = −0.75 [1.54, −0.04], *t* = −2.47, *p* = 0.09, *d* = −0.45, *BF*_10_ = 2.21, but neither comparison reached significance. Yet, the Bayesian statistics indicated the differences were at least anecdotal.

As shown in Figure 2b, the ANOVA shows that the four groups reported differential motivations to participate in the subsequent task in which rewards were no longer provided, *F*(3, 236) = 4.01, *p* = 0.008, $\eta_{p}^{2}$ = 0.05, *BF*_10_ = 3.09. Bonferroni corrected *t*-tests showed that participants’ willingness to participate in the subsequent task was lower in the performance reward group (*M* = 3.83, *SD* = 1.82) than in the monitoring reward group (*M* = 4.97, *SD* = 2.37), difference = −1.13 [−2.13, −0.14], *t* = −2.43, *p* = 0.02, *d* = −0.54, *BF*_10_ = 8.99, lower than in the monitoring group (*M* = 4.77, *SD* = 2.27), difference = −0.93 [−1.93, −0.06], *t* = 2.46, *p* = 0.046, *d* = 0.44, *BF*_10_ = 3.08, and lower than in the baseline group (*M* = 4.98, *SD* = 1.94), difference = −1.15 [−2.15, −0.16], *t* = 2.96, *p* = 0.019, *d* = 0.546, *BF*_10_ = 27.36. There were no detectable difference between the baseline and monitoring groups, difference = 0.22 [−0.78, 1.21], *t* = 0.56, *p* = 1.00, *d* = 0.10, *BF*_10_ = 0.22, between the baseline and monitoring reward groups, difference = 0.02 [−0.98, 1.01], *t* = 0.04, *p* = 1.00, *d* = 0.01, *BF*_10_ = 0.03, and between the monitoring and monitoring reward groups, difference = 0.20 [−1.20, 0.8], *t* = 0.52, *p* = 1.00, *d* = 0.10, *BF*_10_ = 0.03.

Likewise Experiment 1, JOL accuracy was represented by the *G* correlation between predicted performance and actual test performance. Consistent with Experiment 1, JOL accuracy in the JOL reward group (*M* = 0.38 *SD* = 0.87) was significantly greater than that in the monitoring group (*M* = −0.08, *SD* = 0.74), difference = 0.46 [0.16, 0.75], *t*(115) = 3.08, *p* = 0.003, *d* = 0.57, *BF*_10_ = 12.91.

### Discussion

Consistent with Experiment 1, Experiment 2 confirmed the superior effectiveness of monitoring-based rewards over performance-based rewards. However, a notable difference was observed: providing performance-based rewards impaired test performance compared to the performance of non-reward groups. This adverse outcome may be attributed to the higher levels of anxiety associated with math learning compared to other types of learning activities (Ashcraft & Krause, 2007), which likely exacerbated the negative side effects of performance-based rewards.

## 9. General Discussion

In the present study, we introduced a novel paradigm for reward-based learning that emphasizes the alignment between memory performance and the accuracy of metacognitive monitoring, rather than focusing solely on memory performance. This approach led to significant improvements in learning outcomes compared to both the baseline group and traditional performance-based reward group. Moreover, we demonstrated that this effect extends beyond image-pair learning to the transfer of math knowledge, underscoring the robustness of the monitoring-based reward paradigm across various educational materials and tasks. The findings suggest that the mechanism underlying money-motivated memory—where monetary incentives activate the mesolimbic reward system and enhance dopamine release, thereby aiding memory consolidation (see Knowlton & Castel, 2022; Miendlarzewska et al., 2016, for reviews)—remains effective, even when the criteria for earning rewards are refined. Importantly, we demonstrate that neither performance-based rewards nor the act of making JOLs alone can consistently improve learning performance as effectively as monitoring-based rewards. This finding highlights that the combination of accurate metacognitive monitoring and reward is critical for enhancing learning performance and mitigating the negative effects associated with performance-based rewards.

Studies have demonstrated that making judgments of learning (JOLs) can enhance memory performance by promoting elaborative processing and increasing attention to the learning materials (Tauber & Witherby, 2019; Tekin & Roediger, 2020; Zhao et al., 2022). Although these mechanisms were not justified in the present study, in Experiment 1, participants who made JOLs outperformed those in all no-monitoring groups (i.e., baseline and performance reward groups). Additionally, monitoring-based reward, namely associating metacognitive monitoring accuracy and the number of rewards, further improved both memory performance and metacognitive monitoring accuracy. However, in Experiment 2, making JOLs without rewards did not lead to overall performance improvements. This discrepancy may be attributed to the nature of the learning materials used—math theorems—which can make it more challenging to strengthen the cue-target association typically enhanced by JOLs. Additionally, previous studies have suggested that the reactivity effect of making JOLs is typically restricted to word-pair materials (Janes et al., 2018; Li et al., 2022, 2024; Zhao et al., 2022). Therefore, caution is warranted in attributing the benefit of monitor-based rewards solely to the act of making JOLs.

Nonetheless, the provision of rewards in Experiment 2 improved the accuracy of JOLs, potentially aiding students in adaptively adjusting their learning, particularly in self-paced tasks (for a review, see Bjork et al., 2013). Notably, in both experiments, participants demonstrated relatively low—sometimes even negative—metacognitive monitoring accuracy in the pure monitoring group when rewards were absent. However, the accuracy increased significantly with the introduction of rewards. This suggests that rewards may encourage participants to exert greater effort in their judgments of learning. Such an increase in effort not only enhances monitoring accuracy but may also promote more elaborative processing of the materials (Zhao et al., 2022), ultimately improving their learning outcomes.

Consistent with previous studies (e.g., Kuhbandner et al., 2016; Liu et al., 2022), the present study found that performance-contingent rewards did not enhance memory for image-pair learning. This outcome may be attributed to the adverse effects of performance-contingent rewards, which can offset potential benefits despite their interaction with the mesolimbic reward system and the hippocampus. The primary negative consequence of performance-contingent rewards is their tendency to induce pressure-related anxiety, leading to interfering thoughts and superficial encoding (Benware & Deci, 1984; Vansteenkiste et al., 2004; Lepper et al., 2005). Indeed, participants in the performance-reward group reported higher levels of anxiety. Additionally, performance-contingent rewards even led to poorer performance in math knowledge learning, likely because math learning can induce greater anxiety compared to other types of learning activities (Ashcraft & Krause, 2007).

The present study revealed that monitoring-based reward can mitigate performance anxiety, highlighting the positive aspects of monetary incentives. Unlike performance-based rewards, which often drive individuals to pursue extreme outcomes, monitoring-based rewards shift the focus from the fear of failure to the anticipation of potential gains. Furthermore, the compensation for items correctly predicted to be forgotten helps alleviate anxiety related to retrieval failure. Consequently, students in the monitoring-based reward group are less burdened by interfering thoughts and can allocate more cognitive resources to elaborative processing. This is particularly important for making accurate JOLs, as the reward structure incentivizes careful and accurate metacognitive monitoring.

Another concern with providing monetary rewards is the potential undermining of students’ intrinsic motivation for learning (Deci et al., 1999). This study confirmed this well-documented effect, as the performance reward group exhibited decreased motivation for continued learning when the reward was removed, whereas the motivation levels of other groups remained unchanged. Interestingly, despite the provision of monetary rewards, monitoring-based reward did not diminish students’ intrinsic motivation. This may be because the decrease in intrinsic motivation was countered by a positive shift in awareness: students became more cognizant of their achievements, fostering a more positive mindset (Harackiewicz et al., 2000). Thus, the withdrawal of monetary rewards does not necessarily lead to a decline in intrinsic motivation, as the impact of monetary rewards depends on their specific application and the context in which they are used.

## 10. Limitation and Future Directions

One limitation of the present study is that students’ anxiety and intrinsic motivation were measured using a single-item assessment after learning. Although the results from this item supported the expected hypotheses, the effects were not fully conclusive. Future studies could benefit from incorporating real-time physiological measures, such as heart rate and skin conductance, to more accurately reflect students’ anxiety levels during the learning process.

Another limitation of the present study is the lack of item-level learning time data in Experiment 2. While our findings demonstrate that the benefit of monitoring rewards is robust in self-regulated learning, the specific ways in which students monitor and control their learning during this process remain unclear. Future research is needed to address this issue and provide deeper insights.

Additionally, while the effectiveness of monitoring-based reward was demonstrated, the study provided limited insight into its underlying mechanisms. Specifically, neither test anxiety levels nor JOL accuracy were found to correlate strongly with actual learning performance in the present study. This suggests that the benefits of monitoring-based reward may involve multiple components. For instance, in the case of certain materials, such as math knowledge, JOLs alone may not significantly enhance memory performance. Instead, the benefits of monitoring-based reward might stem from other factors, such as reduced anxiety or enhanced covert retrieval processes.

Several hypotheses merit further investigation in future research. First, the advantages of monitoring-based reward may diminish or even disappear with immediate recall tests. This prediction is based on two factors: (1) hippocampal consolidation typically requires time and does not occur immediately during encoding (Murayama & Kuhbandner, 2011), and (2) immediate recall might negate the benefits of covert retrieval practice, which often occurs after learning, particularly when students are aware of their knowledge mastery following explicit JOLs. Thus, monitoring-based reward may be less effective when the interval between learning and testing is minimal.

It would be valuable to investigate whether monitoring-based rewards can also enhance learning outcomes when the total learning time is limited. In both experiments conducted in the present study, the total learning time was fixed, requiring students to allocate their learning time and cognitive resources effectively (Bjork et al., 2013). This constraint may encourage students to focus on easier-to-learn items to maximize efficiency. Conversely, participants in the performance-based reward condition might allocate more time to studying difficult items in an effort to earn greater rewards. This could heighten their anxiety when encountering low-fluency processing of challenging materials, particularly under time pressure (Caviola et al., 2017). However, in the absence of time constraints, students might derive greater benefits from engaging with more difficult items. It remains an open question whether the advantages of monitoring-based rewards would persist under such conditions.

## 11. Educational Implications

In real educational settings, educators often focus on praising students for their excellent performance. However, students who demonstrate strong metacognitive monitoring—i.e., those who can accurately assess their own performance—are frequently overlooked. The present study suggests that, compared to solely rewarding performance, acknowledging and encouraging metacognitive monitoring can offer significant benefits. In everyday learning contexts without external rewards, placing equal importance on metacognitive monitoring alongside performance could be a valuable strategy. This approach may help reduce performance anxiety, encourage more thorough out-of-class review, and foster a growth mindset. By emphasizing the role of metacognitive skills, educators can promote not only better performance but also a more resilient and self-aware approach to learning.

## 12. Conclusion Remarks

Providing students with monetary rewards is often viewed with caution due to its potential negative effects, such as performance-related anxiety and diminished intrinsic motivation. However, the present study demonstrates that it is not the monetary reward itself that leads to these issues, but rather the design of the reward scheme. Specifically, rewarding the alignment between memory performance and metacognitive accuracy resulted in significant memory enhancement and avoided the common pitfalls associated with traditional reward-based learning paradigms. Overall, this study suggests that for educators, metacognitive accuracy should be valued equally alongside pure academic performance. By focusing on how well students can assess their own learning and incorporating reward schemes that reinforce this skill, educators can foster both improved learning outcomes and a healthier learning environment.

## Figures and Tables

**Table 1:** Mean (SD) of reward amount for performance-based and monitor-based groups.

Reward Amount (RMB)	Performance-Based Reward	Monitor-Based Reward
Experiment 1	22.61 (7.27)	33.53 (8.24)
Experiment 2	9.57 (2.24)	11.33 (2.88)

## Data Availability

All the data are available from the OSF link: https://osf.io/3czqr/?view_only=e7d6ea6f9e174b06b794d660e4b1814e (accessed on 16 May 2024).
